# Supplementary material for: Impacts of climate on the biodiversity-productivity relationship in natural forests
Source: Nat Commun. 2018 Dec 21;9:5436. doi: 10.1038/s41467-018-07880-w (PMC6303326; doi:10.1038/s41467-018-07880-w)
Supplement: Supplementary file 1 — Supplementary Information [file 41467_2018_7880_MOESM1_ESM.pdf]

Supplementary Information

Impacts of climate on the biodiversity-productivity relationship in natural forests

Fei et al.

The document contains the following supplementary information:

1. Supplementary Figures 1 and 2
2. Supplementary References

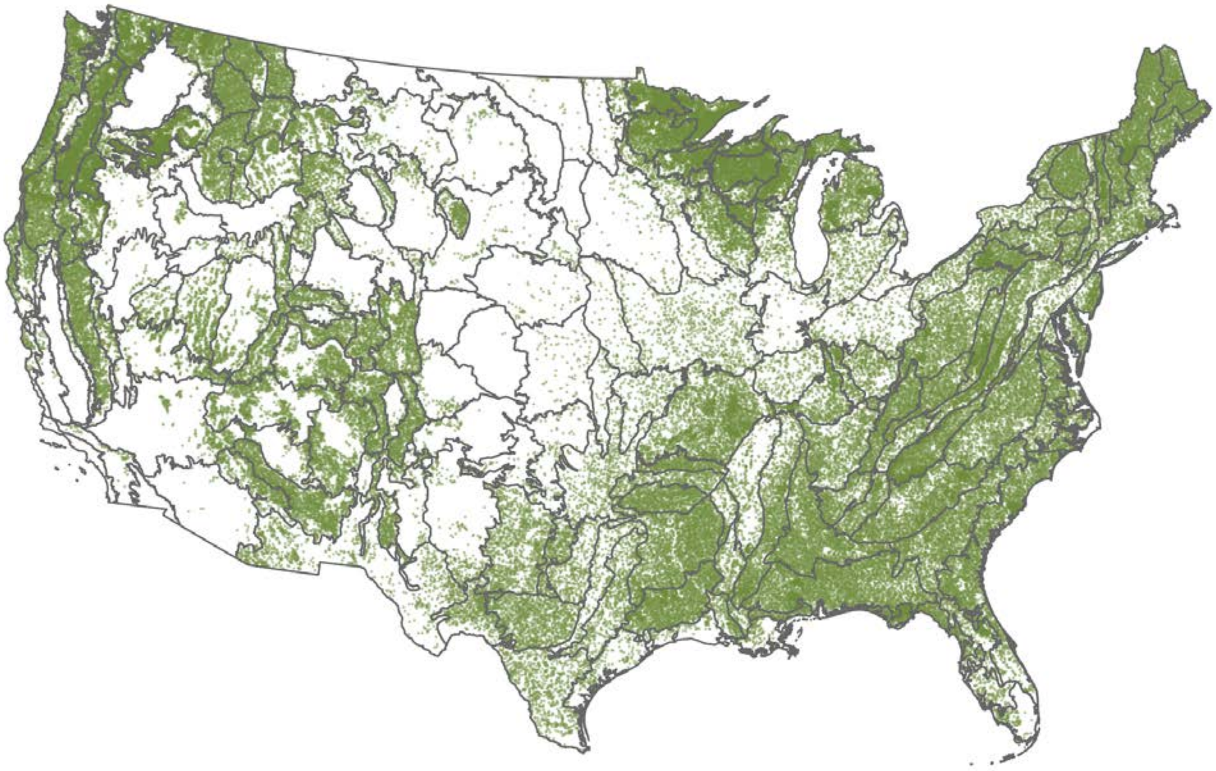

Supplementary Fig. 1. Distribution of forested plots (n = 115,578; each point on the map represents a plot) used in this study with ecoregion boundaries[1] across the contiguous United States. Detailed descriptions about these ecoregions and associated vegetation composition can be found in McNab *et al*[2]. Ecoregion boundaries can be accessed through https://www.fs.fed.us/rm/ecoregions/products/map-ecoregions-united-states/

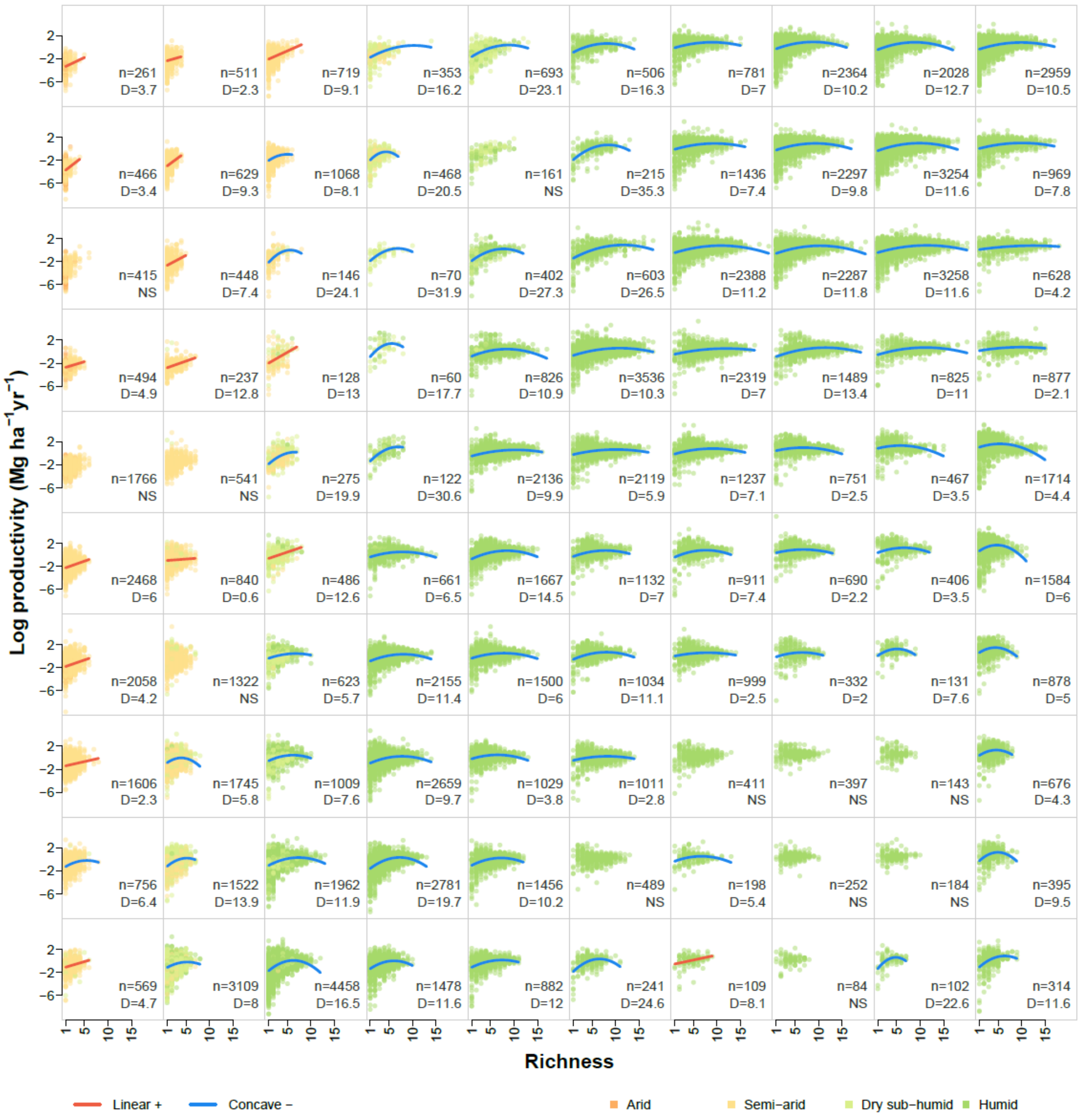

Supplementary Fig. 2. The bivariate relationship between richness and productivity (log-transferred) for each quantile class of the climatic space (see **Fig. 1**). Each plot is colored based on its aridity (arid, semi-arid, dry sub-humid, and humid) according to the Global Aridity Index[3]. Model fitting is based on a generalized linear model (glm) and the best model (linear or quadratic model) is determined based on AIC value. D represents the percentage deviance explained ([[null deviance − residual deviance] / null deviance] × 100) and n is a number of plots within each quantile class. A full list of summary statistics is available in **Supplementary Data File 1**.

Supplementary References

1. Cleland, D. *et al*. Ecological subregions: sections and subsections for the conterminous United States. (USDA Forest Service, 2007).

2. McNab, W. H. *et al*. Description of ecological subregions: sections of the conterminous United States. General Technical Report WO-76B 76, 1-82 (2007).

3. Trabucco, A. & Zomer, R. J. Global aridity index (global-aridity) and global potential evapotranspiration (global-PET) geospatial database (2017).
