# Supplementary material for: Impacts of climate on the biodiversity-productivity relationship in natural forests
Source: Nat Commun. 2018 Dec 21;9:5436. doi: 10.1038/s41467-018-07880-w (PMC6303326; doi:10.1038/s41467-018-07880-w)
Supplement: Supplementary file 3 — Description of Additional Supplementary Files [file 41467_2018_7880_MOESM3_ESM.pdf]

Description of Additional Supplementary Files

File Name: Supplementary Data 1
Description: Results of glm analyses of the relationship between productivity and richness for each climatic quantile class in Fig. 1. *Percentage deviance explained = ((null deviance − residual deviance) / null deviance) × 100

File Name: Supplementary Data 2
Description: Mean posterior coefficient estimates (β1-18) with 95% credible interval in parentheses for the relationships illustrated in Fig. 2a for each climatic quantile class in Fig. 1. *N, normal distribution; Pois, Poisson distribution; μ, mean; σ2, variance; α, intercept; β, slope; non-informative priors were used for intercepts (αs), slope coefficients (βs), and random intercept (μecoregion) from a normal distribution of mean = 0 and variance = 1,000. **An estimate of expected predictive error (lower deviance is better) in JAGS (a program for analysis of Bayesian hierarchical models using Markov Chain Monte Carlo simulation).
